# Mental Health Stigma and Mental Health Literacy in Russia: Their Prevalence and Associations with Somatic, Anxiety, and Depressive Symptoms

**DOI:** 10.11621/pir.2024.0202

**Published:** 2024-06-01

**Authors:** Alena Zolotareva, Natalia Maltseva, Svetlana Belousova, Olga Smirnikova

**Affiliations:** a *HSE University, Moscow, Russia*; b *City Clinical Hospital no. 6, Chelyabinsk, Russia*; c *Chelyabinsk State University, Russia*; d *Sechenov First Moscow State Medical University (Sechenov University), Russia*

**Keywords:** mental health stigma, mental health literacy, somatic symptoms, anxiety symptoms, depressive symptoms

## Abstract

**Background.:**

Mental health stigma and mental health literacy can be potential targets of public education and health development. These areas are culturally specific and have so far been almost unexplored in Russia.

**Objective.:**

This study aimed at examining mental health stigma and mental health literacy in Russia, their prevalence, and their associations with somatic, anxiety, and depressive symptoms.

**Design.:**

The participants were 1,068 Russian adults. They completed the online questionnaire with measures assessing their mental health stigma (Perceived Devaluation and Discrimination Scale; Link et al., 2001); somatic symptoms (Somatic Symptom Scale-8; Gierk et al., 2014); anxiety symptoms (Generalized Anxiety Disorder-7; Spitzer et al., 2006); and depressive symptoms (Patient Health Questionnaire-9; Kroenke et al., 2001). To examine their mental health literacy, we used a series of questions exploring a person’s awareness of mental health and mental health problems.

**Results.:**

Mental health stigma was found in 67% of the participants, who were less confident that most mental disorders can be prevented and more confident that mental disorders can be cured in most cases. Higher devaluation, discrimination, and mental health stigma were related to more severe somatic symptoms. Lower mental health literacy and higher devaluation, discrimination, and mental health stigma were associated with more severe anxiety and depressive symptoms. These associations were the same when adding covariates such as sex, age, partnership, parenthood, and educational background.

**Conclusion.:**

This study highlighted the obvious need for measures to reduce mental health stigma and improve mental health literacy in Russian society. In general, these measures can contribute to the promotion of better mental health in Russia.

## Introduction

The concept of stigmatization dates back to the writings of Erving Goffman. He noted that every society has normative expectations and that people with special characteristics may suffer from stigma, or a label that is deeply discrediting (Goffman, 1963). Initially, scientists believed that stigma is inherent for people with physical and infectious diseases, such as cancer (Huang et al., 2021), visible skin diseases (Germain et al., 2021), HIV, and AIDS (Alonzo & Reynolds, 1995). Further research highlighted that people with mental disorders face mental health stigma, expressed in disgrace, social disapproval, or social discrediting due to their mental health problems (Subu et al., 2021). Shame and embarrassment caused by mental health stigma aggravate the course and outcomes of mental disorders, hinder the sufferer’s search for treatment, and limit the chances of recovery and essential life opportunities (Sartorius, 1998). Mental health stigma may explain the associations between mental disorders and social problems such as social isolation (Jenkins et al., 2023), crime, joblessness, homelessness (Draine et al., 2002), and difficulties in family and intimate relationships (Sell et al., 2021; Hortal-Mas et al., 2022).

Naturally, mental health stigma is the opposite of mental health literacy. Fear and distrust of patients with mental disorders can be combined with ignorance about the causes, treatment, and prevention of mental disorders (Yin et al., 2023). Greater mental health literacy is associated with less public stigma and social distance toward persons with depressive disorders (Svensson & Hansson, 2016); less mental health stigma and less severe stress, anxiety, and depressive symptoms (Tambling et al., 2021); higher intentions to use mental health services; and higher rates of detection of mental disorders (Krakauer et al., 2020). With limited health literacy, people often express false beliefs that mental disorders are contagious and can be explained by an evil spirit, witchcraft, or God’s punishment (Tesfaye et al., 2021).

Mental health stigma and mental health literacy have cultural specificity and should be examined with respect to particular cultures and countries (Krendl et al., 2020; Ran et al., 2021; Vovou et al., 2021). Previous studies have shown that Russian participants are less tolerant of people with mental disorders than British respondents (Shulman & Adams, 2002), and more often than American participants, label people with mental disorders as “weak-willed” and leading an “immoral lifestyle” (Nersessova et al., 2019). Russian youth with affective disorders admitted that they have refused medical care and treatment for fear of a negative reaction and labeling from people in their social environment (Cantarero-Arévalo et al., 2020). Considering the obvious burden of mental health stigma and limited mental health literacy in Russia, as well as the lack of existing evidence of the relationship between mental health stigma, mental health literacy, and mental health problems (Alonso et al., 2008; Guo et al., 2023; Haruyama et al., 2022), this study aimed at examining mental health stigma and mental health literacy in Russia, and their prevalence and associations with somatic, anxiety, and depressive symptoms.

## Methods

### Participants

The data were collected from June to November 2023. We distributed a link to an electronic questionnaire on social networks (Telegram, VKontakte) and invited Russians 18 years and older to take part in the study. There were 1,110 participants, but 32 respondents were excluded due to their failing to complete the questionnaire. The participants were 1,068 Russian respondents, including 673 women and 395 men age 18 to 50 years (M = 21.43, SD = 6.13). Most of the participants had not received any higher education (n = 673; 63%), were in a marital or romantic relationship (n = 545; 51%), and did not have children (n = 989; 93%).

### Measures

The participants completed questionnaires measuring their degree of mental health stigma, mental health literacy, and mental health problems.

Mental health stigma was examined with the Perceived Devaluation and Discrimination Scale (PDD; Link et al., 2001). The PDD is a 12-item measure assessing the beliefs that others will devalue or discriminate against a person with a mental disorder. The items are rated on a four-point Likert scale ranging from 0 (“strongly disagree”) to 3 (“strongly agree”). Mental health stigma is considered high with a score of 2.5 or more (Brohan et al., 2010). We used the translated Russian version of the PDD. In this study, the Cronbach’s alpha was .718.

Mental health literacy was examined with a series of questions assessing a person’s awareness of mental health and mental health problems. The respondents had to agree (“yes”) or disagree (“no”) with the following statements: “Mental health is linked to a healthy lifestyle;” “Stress is not the cause of all mental disorders;” “Mental disorders can be cured in most cases;” “Most mental disorders can be prevented;” and “Mood stability is one of the signs of mental health.” The higher the sum of positive responses, the higher the score on mental health literacy.

Somatic symptoms were examined with the Somatic Symptom Scale-8 (SSS-8; Gierk et al., 2014). The SSS-8 is an 8-item measure assessing burden of somatic symptoms during the past seven days (*i.e.,* “stomach or bowel problems,” “pain in arms, legs, or joints,” “feeling tired or having low energy”). The items are rated on a five-point Likert scale ranging from 0 (“not at all”) to 4 (“very much”). We used the Russian version of the SSS-8 (Zolotareva, 2022). In this study, the Cronbach’s alpha was .820.

Anxiety symptoms were examined with the Generalized Anxiety Disorder-7 (GAD-7; Spitzer et al., 2006). The GAD-7 is a 7-item measure evaluating burden of anxiety symptoms during the past two weeks (*i.e.,* “trouble relaxing,” “not being able to stop or control worrying”). The items are rated on a four-point Likert scale ranging from 0 (“not at all”) to 3 (“nearly every day”). We used the Russian version of the GAD-7 (Zolotareva et al., 2023a). In this study, the Cronbach’s alpha was .910.

Depressive symptoms were examined with the Patient Health Questionnaire-9 (PHQ-9; Kroenke et al., 2001). The PHQ-9 is a 9-item measure assessing burden of depressive symptoms during the past two weeks (i.e., “poor appetite or overeating,” “little interest or pleasure in doing things”). The items are rated on a four-point Likert scale ranging from 0 (“not at all”) to 3 (“nearly every day”). We used the Russian version of the PHQ-9 (Zolotareva et al., 2023b). In this study, the Cronbach’s alpha was .876.

### Data analysis

The data were analyzed using SPSS for Windows v. 27.0. Since the participants who did not fully complete the questionnaire were excluded, there were no missing data. Means and standard deviations for continuous variables and frequencies, and percentages for categorical variables, were calculated to describe the participants’ characteristics and the prevalence of their mental health stigma and mental health literacy. We used Pearson’s χ^2^ test to assess differences in mental health literacy based on low and high mental health stigma. Multiple linear regression analyses by forced inclusion were performed to examine how independent variables (mental health stigma and mental health literacy) predicted the dependent variables (somatic, anxiety, and depressive symptoms).

## Results

The prevalence of mental health stigma was 67%. *Table 1* presents the prevalence of mental health literacy. The participants with low mental health stigma were less confident that most mental disorders can be prevented (χ^2^ (1) = 8.671, p = .003) and more confident that mental disorders can be cured in most cases (χ^2^ (1) = 9.574, p = .002) than participants with mental health stigma. There was no difference in the participants’ agreement that mental health is linked to a healthy lifestyle (χ^2^ (1) = .111, p = .739), that stress is not the cause of all mental disorders (χ^2^ (1) = 3.466, p = .063), and that mood stability is one of the signs of mental health (χ^2^ (1) = .167, p = .683).

**Table 1:** Prevalence of mental health literacy

Items	Total	Low MHS	Hight MHS
1 Mental health is linked to a healthy lifestyle	85%	85%	86%
2 Stress is not the cause of all mental disorders	60%	55%	61%
3 Mental disorders can be cured in most cases	72%	78%	69%
4 Most mental disorders can be prevented	79%	74%	81%
5 Stable mood is one of the signs of mental health	86%	86%	85%

*Note. MHS = mental health stigma. The cut-off score of PDD ≥ 2.5 shows high MHS.*

*Table 2* illustrates the results of the linear regression analyses. Higher devaluation, discrimination, and mental health stigma predicted higher somatic symptoms (R^2^ = .013). Lower mental health literacy and higher devaluation, discrimination, and mental health stigma predicted higher anxiety symptoms (R^2^ = .030). Similarly, lower mental health literacy and higher devaluation, discrimination, and mental health stigma predicted higher depressive symptoms (R^2^ = .037). These patterns were the same when adding the covariates for somatic symptoms (R^2^ = .084), anxiety symptoms (R^2^ = .083), and depressive symptoms (R^2^ = .092).

**Table 2:** Results of the linear regression analyses

	Somatic symptoms	Anxiety symptoms	Depressive symptoms
	β (95% CI)	β (95% CI)	β (95% CI)
Unadjusted model			
Devaluation	.212*	.271**	.355***
Discrimination	.259***	.287***	.356***
Mental health stigma	.372***	.380***	.503***
Mental health literacy	–.038	–.099**	–.080**
Adjusted model			
Devaluation	.200*	.258**	.338***
Discrimination	.235***	.265***	.338***
Mental health stigma	.327***	.335****	.459***
Mental health literacy	–.042	–.101**	–.085**

Note. The following covariates were included in the adjusted model: sex, age, partnership, parenthood, education background. * p < .05; ** p < .01; *** p < .001.

## Discussion

This study aimed to examine mental health stigma and mental health literacy in Russia, their prevalence, and their associations with somatic, anxiety, and depressive symptoms. Some key findings can be identified.

First, both mental health stigma and mental health literacy were common in this Russian sample. The prevalence of mental health stigma was 67%, which is similar to the prevalence of mental health stigma in 75% of the South Indian population (Venkatesh et al., 2015) and is much higher than the prevalence of self-stigma in people living with mental illness (29%) (Alemu et al., 2023) and in caregivers of children and adolescents with mental illness (39%) (Minichil et al., 2021). There is reason to suppose that people who know the nature of mental illness are less likely to stigmatize persons with mental disorders. In 60-86% of cases, our sample gave correct answers to questions about mental health and mental disorders. These values coincide with the prevalence of mental health literacy in university students in Bangladesh (62%) (Siddique et al., 2022) and in people living in Ethiopian communities (55%) (Tesfaye et al., 2021).

Second, Russians with mental health stigma were less confident that mental disorders can be cured and more confident that most mental disorders can be prevented. This means that people with mental health stigma blame patients with mental disorders for not preventing their mental health disorders and coheres with the fact that self-blame is associated with psychological distress in patients with mental health disorders (Jannati et al., 2020). Conversely, people with low mental health stigma generally believe that patients with mental disorders can recover under certain circumstances. This belief is realistic, because studies have shown that 10% of patients with a lifetime history of psychopathology have reported optimal psychological functioning, and 5% of patients with suicidal ideation, 6% of patients with anxiety disorders, and 7% of patients with depressive disorders experienced optimal well-being following recovery after mental disorders (Devendorf et al., 2022).

Third, higher mental health stigma, but not lower mental health literacy was associated with higher somatic symptoms. Public stigma is higher for patients with depression than for patients with symptoms of somatic symptom disorder, regardless of the type of symptom and existence of an earlier somatic disorders (von dem Knesebeck et al., 2018). Although people often recognize the psychological nature of their somatic symptoms and do not try to hide or “mask” their psychological ill-health and discomfort (Skapinakis & Araya, 2011), in many cultures somatic disease is justified, and mental illness is condemned. Therefore, somatization has cultural value and social effectiveness, helping a person avoid public stigma and labels of mental disorders (Kleiman, 1986). This explains the positive association between public stigma and somatic symptoms (McNealy & Lombardero, 2020).

Fourth, higher mental health stigma and lower mental health literacy were associated with higher anxiety and depressive symptoms. This result coheres with those from other studies. For example, suffering from anxiety or depressive symptoms almost doubles mental health stigma, and the comorbidity of anxiety and depressive symptoms further increases these associations according to Alonso et al. (2008). Regarding the Saudi population, studies have found that 88% of people had lack of mental health literacy, 59% expressed a negative perception of mental illness, 67% reported negative attitudes toward mentally ill patients, and 55% experienced negative attitudes toward professional help-seeking (Abolfotouh et al., 2018). These findings also determined that mental health literacy interventions reduce mental health stigma (Ma et al., 2023) as well as anxiety and depressive symptoms (Magallón-Botaya et al., 2023), improve competencies for maintaining mental health, and encourage seeking help in the case of mental health problems (Beukema et al., 2022).

In general, the high prevalence of mental health stigma and limited mental health literacy, and their close relationship with somatic, anxiety, and depressive symptoms in Russians may have a deep cultural and historical roots. In Medieval Russia, the holy fool was celebrated as someone who refused earthly comforts and told the truth about what was happening, but in the 18th century the holy fools were locked up in “yellow houses” because they were considered crazy and socially dangerous (Brintlinger & Vinitsky, 2007). Russian psychiatry, which has had a dramatic history since the 18th century, despite social, economic, and political diffculties, is currently actively developing and humanizing (Krasnov & Gurovich, 2012). Well-designed cultural and historical features can influence Russians even in the context of the current state of psychiatry and the revival of a humane societal attitude toward patients with mental disorders.

## Conclusion

To our knowledge, ours is the first study highlighting the prevalence of mental health stigma and mental health literacy in Russia. We found that the majority of Russians in our sample had mental health stigma and limited mental health literacy. In addition, we showed that mental health stigma and limited mental health literacy were associated with mental health problems such as somatic, anxiety, and depressive symptoms. These findings may determine the prospects for a closer examination of mental health stigma, mental health literacy, and mental health problems in Russia.

## Limitations

This study had a number of limitations. Its cross-sectional nature limits judgments about causal relationships between mental health stigma, mental health literacy, and mental health problems. Furthermore, the participants were mostly young women, which may distort the findings. Previous research has shown that women and younger people show less stigmatization and more literacy about mental health issues than men (Chandra et al., 2006; Hadjimina & Furnham, 2017; Ricciardelli et al., 2021). Finally, mental health literacy was measured using small questions and should probably be studied more thoroughly in future research.

## Clinical implications

The findings of this study highlight the obvious need for measures to reduce mental health stigma and improve mental health literacy in Russia. Traditional anti-stigma campaigns have focused on raising mental health literacy, but recently scientists have identified equally important strategies such as expanding social contacts, advocacy by influential figures or groups, and the enactment of anti-discriminatory laws (Shahwan et al., 2022). Considering the fact that somatic, anxiety, and depressive symptoms appear to be more common to people with mental health stigma and limited mental health literacy, such campaigns can contribute to the promotion of better mental health in Russia.
